# Comparative study on cellular entry of incinerated ancient gold particles (Swarna Bhasma) and chemically synthesized gold particles

**DOI:** 10.1038/s41598-017-10872-3

**Published:** 2017-09-06

**Authors:** Daniel Beaudet, Simona Badilescu, Kiran Kuruvinashetti, Ahmad Sohrabi Kashani, Dilan Jaunky, Sylvie Ouellette, Alisa Piekny, Muthukumaran Packirisamy

**Affiliations:** 1 Department of Biology, Concordia University, Montreal, Quebec H4B 1R6 Canada; 2 Department of Mechanical and Industrial Engineering, Concordia University, Montreal, Quebec H3G 1M8 Canada

## Abstract

Gold nanoparticles (AuNPs) are used for a number of imaging and therapeutic applications in east and western part of the world. For thousands of years, the traditional Indian Ayurvedic approach to healing involves the use of incinerated gold ash, prepared with a variety of plant extracts and minerals depending on the region. Here, we describe the characterization of incinerated gold particles (IAuPs) in HeLa (human cells derived from cervical cancer) and HFF-1 (human foreskin fibroblast cells) in comparison to synthesized citrate-capped gold nanoparticles (AuNPs). We found that while individual IAuP crystallites are around 60 nm in size, they form large aggregates with a mean diameter of 4711.7 nm, some of which can enter cells. Fewer cells appeared to have IAuPs compared to AuNPs, although neither type of particle was toxic to cells. Imaging studies revealed that IAuPs were in vesicles, cytosol, or in the nucleus. We found that their nuclear accumulation likely occurred after nuclear envelope breakdown during cell division. We also found that larger IAuPs entered cells via macropinocytosis, while smaller particles entered via clathrin-dependent receptor-mediated endocytosis.

## Introduction

Gold nanoparticles (AuNPs) are used for a number of imaging and therapeutic applications including diagnosis and treatment of cancers^1, 2^. Their physical and chemical properties are tunable, as they strongly depend on size, shape, aggregation state, and surface chemistry^3^. The use of AuNPs in modern medicine can be traced back to their use in the ancient traditional Indian Ayuverdic approach to healing^4–7^ of many ailments. Ayuverdic (where Ayus means life principle, and veda refers to system of knowledge) is a philosophy, concerned with the protection of “ayus”, by combining healthy living with therapeutic measures. Medicinal preparations typically consist of mixtures of plant- and animal -derived products, minerals and other metals^6, 8, 9^. Gold derived Ayuverdic medicine is called Swarna Bhasma (gold ash)^4, 10–12^, and testing their localization, entry and impact on human cells in comparison to chemically synthesized AuNPs will be the focus of this study.

Swarna Bhasma gold ash is prepared through a process called Putapaka, which involves heating and quenching gold with various plant extracts. Gold is hammered into a ribbon from a coarse powder, then ground with various herbal extracts and incinerated at high temperature (∼1000 °*C*) in earthen crucibles. During the incineration phase, which is repeated several times, the size of gold particles is reduced more and more with each cycle, via mechanical comminution^10, 13^. It is important to note that by this top down approach, the gradual reduction in size of the gold particles brings them increasingly closer to colloidal AuNPs. The gold ash, Swarna Bhasma, prepared according to Ayuverda texts will be called Incinerated Gold Particles (IAuPs) for this study.

Several comparisons have been made between IAuPs and colloidal AuNPs, which are chemically synthesized through the reduction of gold salts by various natural or chemical reducing agents^14^. Chemically synthesized AuNPs can be made with different surfactants and stabilizing groups, and their size can vary from 1 to 100 nm. Spectroscopic measurements of IAuPs revealed that they are comprised of individual particles of around 50–70 nm in size. Unlike chemically synthesized AuNPs, they are not well separated and form large aggregates over 2 μm in size^13, 15, 16^. The composition and size of IAuPs from different pharmaceutical companies is variable. They contain a range of compounds and elements, including heavy metals^17–19^, derived from herbal extracts typically used for medicinal purposes. The presence of heavy metals has been associated with possible contamination of the soil, where the plants are grown, or from the crucibles used for the long calcination processes. Thus, the size, composition and morphology of AuNPs and IAuPs are different. However, given that both are used in medical applications, it is crucial to understand how they interact with, and impact, the core physiological functions of human cells^1, 14, 20^.

Several studies have explored the entry mechanisms and cytotoxicity of colloidal AuNPs with different surface moieties, size and morphology *in vitro*, with variable outcomes. In general, small spherical particles (e.g. <2 *nm*) were reported to cause cytotoxicity in different mammalian cell lines, when compared to rod shaped particles, although their toxicity varied depending on the surface coating (cationic vs anionic) and the cell line^14, 21^. It is assumed that most AuNPs less than 50 nm in size enter cells via receptor-mediated endocytosis in a clathrin-dependent manner^21, 22^. However, evidence suggests that they also enter cells via caveolin-mediated endocytosis or macropinocytosis^23–25^ depending on their size, shape, surface coating, and if they form aggregates^1, 26–29^. Also, they could enter via these different pathways depending on the cell type and/or receptors expressed at their surface^1, 21, 28, 29^. It may also be desirable to target AuNPs to the cytosol or other subcellular locations inside the cell, such as the nucleus or mitochondria. However, to do this, they must escape from the endomembrane system, since particles are initially contained in vesicles, regardless of the mechanism of entry^30^. Surface functionalization could promote their escape from lysosomes into the cytosol, as in the case of encapsulation of AuNPs in a cationic core-shell polymer colloid that expands upon acidification in the lysosome causing rupture^31^, while larger aggregates could mechanically disrupt vesicle membranes. There are reports of using protein tags with specific amino acid sequences known to mediate transport into the nucleus^32^ or mitochondria^33, 34^. Again, it is not clear how these tags have accessibility to the protein complexes that recognize them when the particles are retained in vesicles, particularly when they are in lysosomes or autophagosomes. However, once they are in the cytosol, the tags could become accessible to mediate transport. In addition, very small particles may not require tags to pass through the nuclear pores, or outer mitochondrial membrane.

This paper presents a comparative study of AuNPs and IAuPs in their (i) localization, (ii) physiological impact and (iii) entry in cancerous and non-cancerous human cells, which are crucial to understand for their design and therapeutic use. We found that IAuPs contain a large variety of elements, some of which are present at significant concentrations (e.g. Mg and Ca). While individual IAuP crystallites are 60 nm in size, they form large aggregates with a mean diameter of 4711.7 nm. IAuPs and AuNPs imparted no obvious toxicity to HeLa cells (human cervix adenocarcinoma) or HFF-1 cells (human foreskin fibroblasts, which are non-cancerous). Imaging revealed that while some IAuPs were in membrane-bound vesicles or vacuoles, others were in the cytosol and nuclei of cells, while AuNPs accumulated primarily in the endomembrane system. Mechanically disrupting IAuPs into smaller 100–200 nm particles increased their accumulation in cells where they localized to the endomembrane system similar to AuNPs. Interestingly, larger IAuPs accumulated in the nuclei of HeLa cells after nuclear envelope breakdown during cell division. Further studies revealed that IAuPs enter cells by more than one mechanism, as their entry was reduced after treatment with Cytochalasin D to block macropinocytosis, and Chlorpromazine to block clathrin-mediated receptor-mediated endocytosis. These studies show that IAuPs are large, inert aggregates, which could be explored for use as carriers.

## Materials and Methods

### Synthesis and Characterization of IAuPs and AuNPs

Citrate-capped spherical AuNPs were prepared by the reduction of chloroauric acid with sodium citrate using the Turkevich method^35, 36^. Briefly 75 mL of chloroauric acid solution containing 45 μ*g*/*ml* gold was heated, and 5 mL of 1% sodium citrate was added to the boiling solution. After the solution turned purple, it was boiled for another 15 minutes, then left to cool to room temperature. The elemental composition of the synthesized AuNPs was measured by ICP-MS (Inductively-Coupled Plasma Mass Spectroscopy) on a 7700x Agilent and Energy Dispersive Spectroscopy (EDS)-SEM, and the shape and size of the AuNPs was determined by SEM using the Hitachi S 3400N.

**Figure 1:**
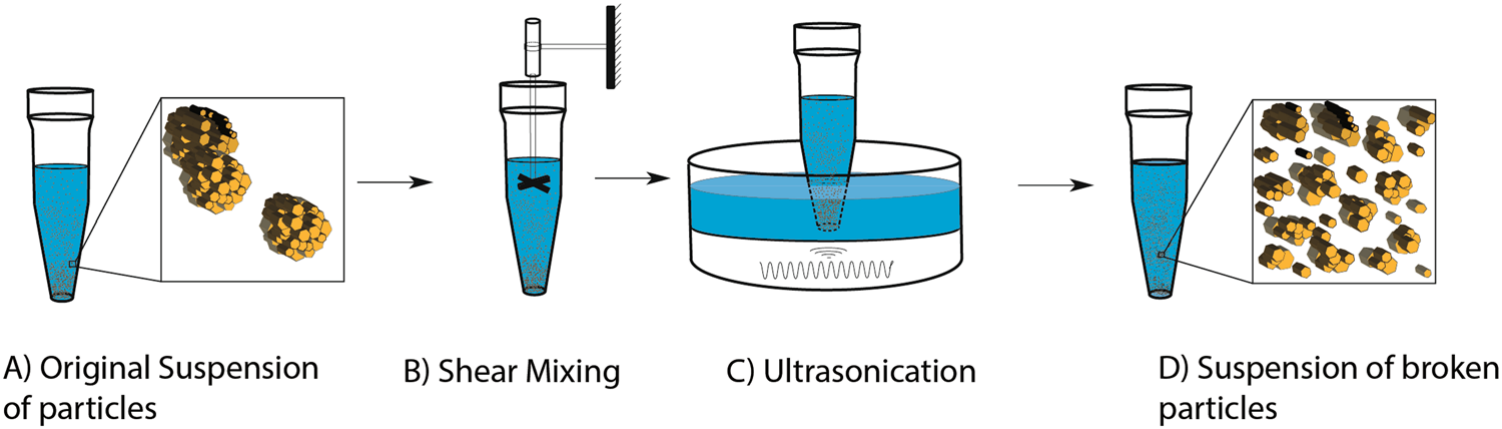
A Schematic diagram illustrating the process of mechanically disrupting IAuPs. IAuPs suspended in deionized water are broken with a homogenizer (Step 1) followed by ultrasound treatment (Step 2) to break apart larger particles into smaller particles.

IAuPs were obtained as a powder from Jaya Indian Medicine Pharmaceutical Pvt Ltd, Maduravoyal, Chennai, Tamilnadu, India. IAuPs were suspended in de-ionized water for use in all experiments. To determine particle size and shape, IAuPs were dried on pre-cleaned glass slides at room temperature and imaged by SEM using the Hitachi S 3400N. In addition, Dynamic Light Scattering (DLS) measurements were done using a Nicomb 380 instrument by Dynalene Lab Services. The elemental composition of IAuPs was measured using EDS-SEM and ICP-MS. For ICP-MS, the sample was oven dried and digested with aqua regia (3HCL: 1HNO3) at 110 °C for 3 hours. Then the sample was cooled and filtered through a 0.2 μ*m* PTFE filter. The sample was analyzed using “No gas” and “He” modes. To break the IAuPs into smaller particles, IAuPs in de-ionized water were broken mechanically (using Omni Mixer Homogenizer 20–25 minutes at a variable speed), and subsequently by ultrasound treatment (using Branson 200 Ultrasonic cleaner at 40 kHz, 8–10 times and 5 minutes each time) (Fig. 1).

### Cell Culture

HeLa (human cervix adenocarcinoma) and HFF-1 (human foreskin fibrobast) cells were cultured in Dulbecco’s modified Eagle’s Medium (DMEM; Wisent) supplemented with 10 or 15% Fetal Bovine Serum (FBS; ThermoFisher Scientific), 100 U penicillin and 0.1 mg/mL streptomycin (Wisent), and 2 mM L-glutamine (Wisent). Cells were incubated at 37 °C in a humidified chamber with 5% CO_2_, and passaged at 75–100% confluency or as needed for the analysis of IAuPs or AuNPs in cells.

### Immunoflourescence and Microscopy

Cells were fixed for immunofluorescence using 10% trichloroacetic acid (TCA) as previously described^37^. Fixed cells were immunostained for microtubules using 1:250 mouse anti-tubulin antibodies (DM1A, Sigma-Aldrich) and anti-mouse Alexa 488 secondary antibodies were used at a 1:400 dilution. DAPI (Sigma-Aldrich) was added at a 1:1000 dilution (1 mg/mL stock) for 5 minutes before mounting the coverslips onto slides. Fixed cells were imaged using a Leica DMI6000B epifluorescence microscope with the 63x/1.4 PL APO oil immersion objective (pixel size 0.102 μ*m*), and z-stacks of 0.5 μ*m* were acquired with a Hamamatsu OrcaR2 camera and Volocity software (PerkinElmer) using a piezo Z stage (MadCityLabs). Image files were exported as TIFFs, which were converted into maximum intensity z-stack projections in Image J (NIH). Hyperspectral microscopy was performed using the Cytoviva hyperspectral imaging system with enhanced darkfield optical illumination (Cytoviva, Inc) on AuNPs or IAuPs in HeLa cells. Using the 60x/1.4 or 100x/1.4 oil objectives, dark-field images were collected at oblique angles, and the reflective fluorescence was measured for selected pixels, using a spectrophotometer integrated CCD, with a spectral range of 400–1000 nm and spectral resolution of 2.8 nm.

Samples for SEM were fixed as above, but then were washed 3–4 times with TBST wash buffer (50 mM Tris pH 7, 150 mM NaCl, 0.5% Triton-X 100) and dehydrated, using a series of solutions ranging from 50–100% ethanol. Dehydrated samples were covered and placed in a fume hood overnight for complete drying. The cells were imaged using SEM (Hitachi S 3400 N) under a voltage of 15 kV and vacuum of 50 Pa, and images were collected after zooming in as indicated.

To perform live imaging, media was replaced with phenol red-free media. Cells were plated on 25 mm round coverslips (No. 1.5), placed in a 35 mm chamlide magnetic chamber (Quorum) and kept at 37 °C with 5% CO_2_ using the INU-TiZ-F1 chamber (MadCityLabs). Cells were treated with either IAuPs or citrate-capped AuNPs for 24–48 hours prior to imaging. Cell membranes were stained using FM 4–64 lipophilic Styryl Dye (Invitrogen) 20–60 minutes prior to image acquisition. Live imaging was performed on an inverted Nikon TiE microscope using the 100x Plan APO WD oil immersion objective, and an Evolve (Photometrics) EMCCD camera using Elements 4.0 acquisition software (Nikon). Images were acquired with 30 ms exposures for brightfield, and 100 ms for fluorescence using the Heliophore LED with an excitation wavelength of 480 nm at 15% power (National Instruments). Z-stacks of 0.5 μ*m* thickness were collected using a NI-DAQ piezo Z stage (National Instruments) every 3 seconds. Image files were exported as TIFFs, which were opened with Image J (NIH) and converted into maximum intensity z-stack projections.

### Nuclear Entry Assay

To test for nuclear entry of the IAuPs, HeLa cells were blocked in S phase of the cell cycle using Thymidine as described previously^37^. A ‘double’ block was performed to ensure that the entire cell population was synchronized to be in S phase. To do this, HeLa cells were plated at 30–40% confluency on glass coverslips. They were treated with 2 mM Thymidine (Sigma) for 16 hours, then released for 8 hours after washing 3 X with PBS phosphate buffered saline (pH 7.4; 1.06 mM KH2PO4, 154 mM NaCl, 5.6 mM Na2HPO4), and adding and pre-warmed media. Cells were treated again with 2 mM Thymidine and after 1 hour, 200 μ*g* of IAuPs was added to the cells. After 23 hours, the cells were fixed and immunostained as described above, and brightfield and fluorescence microscopy were used to assess localization of the IAuPs. Control cells did not receive the second Thymidine treatment to keep them cycling. This experiment was replicated for statistical analysis.

### Cellular Entry Assay

To determine the mechanism by which IAuPs or AuNPs enter cells, HeLa cells were pre-treated with (i) 1% DMSO as control, (ii) 100 nM Cytochalasin D to block F-actin and disrupt macropinocytosis, (iii) 100 μ*m* Genistein to block caveolin-mediated endocytosis, or (iv) 5 μ*m* Chlorpromazine to block clathrin-dependent receptor-mediated endocytosis. HeLa cells were plated at 30–40% confluency on glass coverslips, and 100  μ*g* IAuPs were added one hour after adding the drugs. After another 7 hours, cells were fixed and immunostained as described above for brightfield and fluorescence microscopy to assess localization of IAuPs. All treatments were replicated for statistical analysis.

### Analysis

To determine the proportion of cells with IAuPs located in the nucleus, a minimum of 15 fields of view covering around 300–400 cells were imaged, and only cells with IAuPs were counted. Both the brighfield and DAPI images for multiple z-panes were used to access nuclear localization. Averages and standard deviations were calculated. To assess the entry mechanism of IAuPs, the total proportion of cells containing IAuPs was determined using a minimum of 10 fields of view covering 150–200 cells imaged per treatment. Averages and standard deviations were calculated and graphed. The student t-test was used to determine the statistical significance (*p* < 0.05).

## Results

### Physicochemical characterization of Incinerated Gold Particles (IAuPs)

**Figure 2:**
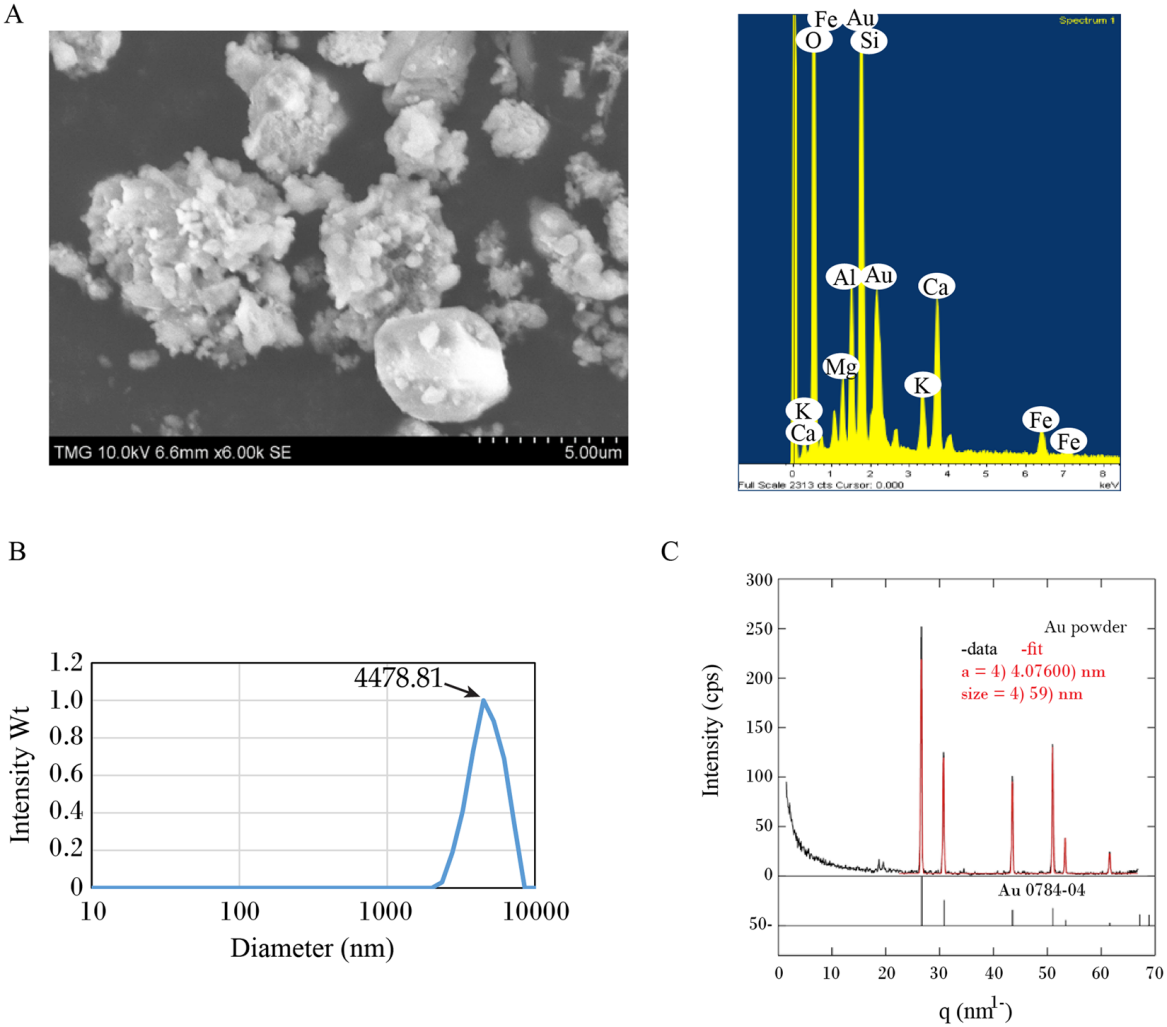
(**A**) IAuPs were imaged by SEM (left) and by EDS-SEM (right), which shows the shape and size of the particles, and the elemental composition, respectively. (**B**) A graph shows the mean size of the IAuPs by DLS. (**C**) A graph shows the XRD pattern of IAuPs in comparison to Au, and the size of individual particles is indicated.

Since the preparation and composition of Swarna Bhasma IAuPs can vary depending on the geographical region, the IAuPs used in this study were characterized *in vitro* ^15–19^. DLS analysis revealed that IAuPs have a broad range in size with a mean diameter of 4711.7 nm (Fig. 2B). SEM imaging also showed the variation in size and irregular morphology of the particles (Fig. 2A). Further analysis of IAuPs using X-ray diffraction revealed that the size of crystallites within the IAuPs is approximately 60 nm (Fig. 2C). Therefore, IAuPs are likely large aggregates of smaller nanoparticles.

The composition of IAuPs in powder form was determined using EDS-SEM and ICP-MS. As shown in Table 1 and Fig. 2A, gold was found to be the most abundant element in IAuPs. Interestingly, this value was low in comparison to other samples that have been characterized^13, 15–19^. ICP-MS revealed that the particles contained other elements, some of which were present at higher concentrations, such as Mg, Ca, Fe, and Si (0.29–1.9%), while others (Mn, Ni, As) were found in trace amounts (Table 1). This was consistent with EDS-SEM results, which showed that the concentration of gold appeared to vary depending on the area used for analysis, with significant peaks corresponding to elements including Mg, Ca, Fe, and Si (Fig. 2A). Additional components in the IAuPs are likely oxygen and carbon caused by oxidation during the incineration process. The presence of oxygen in bhasmas was revealed by X-ray fluorescence spectroscopy^14^.

**Table 1:** The elemental composition of IAuPs determined by ICP-MS.

*Elements*	*Concentration %*
Gold (Au)	56.88
Mg	1.8
Ca	1.4
Fe	0.29
Si	0.29
**Trace Elements**	
Mn	0.037
Ni	0.02
As	0.15

**Figure 3:**
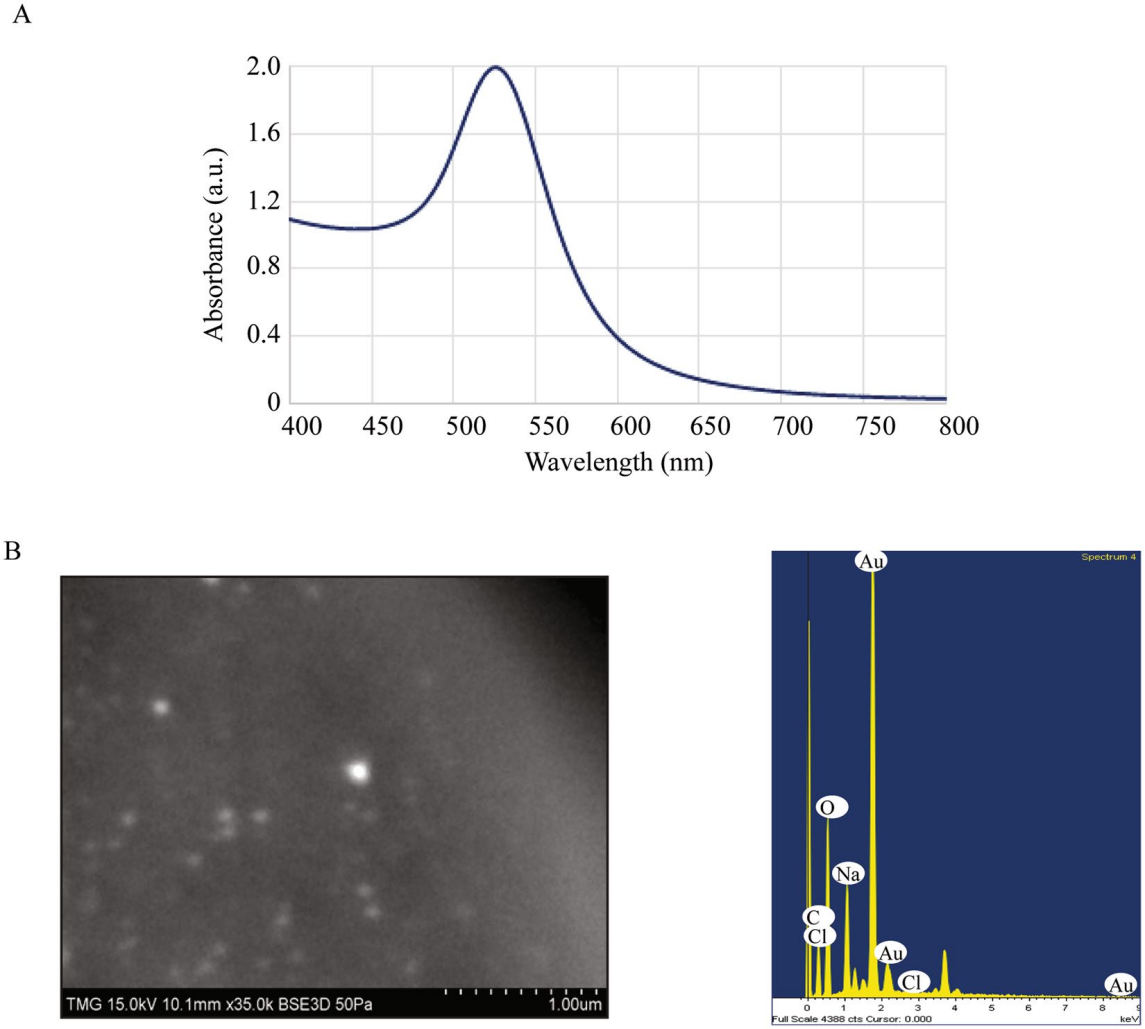
(**A**) The graph shows the LSPR band corresponding to AuNPs. (**B**) AuNPs were imaged by SEM (left) and by EDS-SEM (right), which shows the shape and size of the particles, and the elemental composition, respectively.

The synthesized gold nanoparticles (AuNPs) generated for this study to compare cellular toxicity and localization with IAuPs was characterized *in vitro*. The size of the AuNPs was estimated from the UV-Visible spectrum of the colloidal solution (Fig. 3A). Their diameter was calculated using the following formula^38^.

1 $$d=\frac{ln(\frac{{\lambda }_{SPR}-{\lambda }_{0}}{{L}_{1}})}{{L}_{2}}$$

Where ${\lambda }_{SPR}$ is the position of the band in the spectrum of the solution, ${\lambda }_{0}=512nm$ (Fig. 3A), ${L}_{1}=6.53$ and ${L}_{2}=0.0216$ are the fit parameters determined from the theoretical values. The average diameter of the colloidal AuNPs was calculated to be 32 nm. The elemental composition of colloidal AuNPs as determined by ICP-MS and EDS revealed that gold was the most abundant element as expected (Table 2; Fig. 3B). Imaging AuNP by SEM showed that they are spherical and may vary in size up to 100–200 nm (Fig. 3B).

**Table 2:** The elemental composition of synthesized AuNPs determined by ICP-MS.

*Elements*	*Concentration (ppm)*
Gold (Au)	89.6
Mg	0.273
Ca	1.16
Na	20.9
Si	2.69

### Characterization of IAuPs in human cells

**Figure 4:**
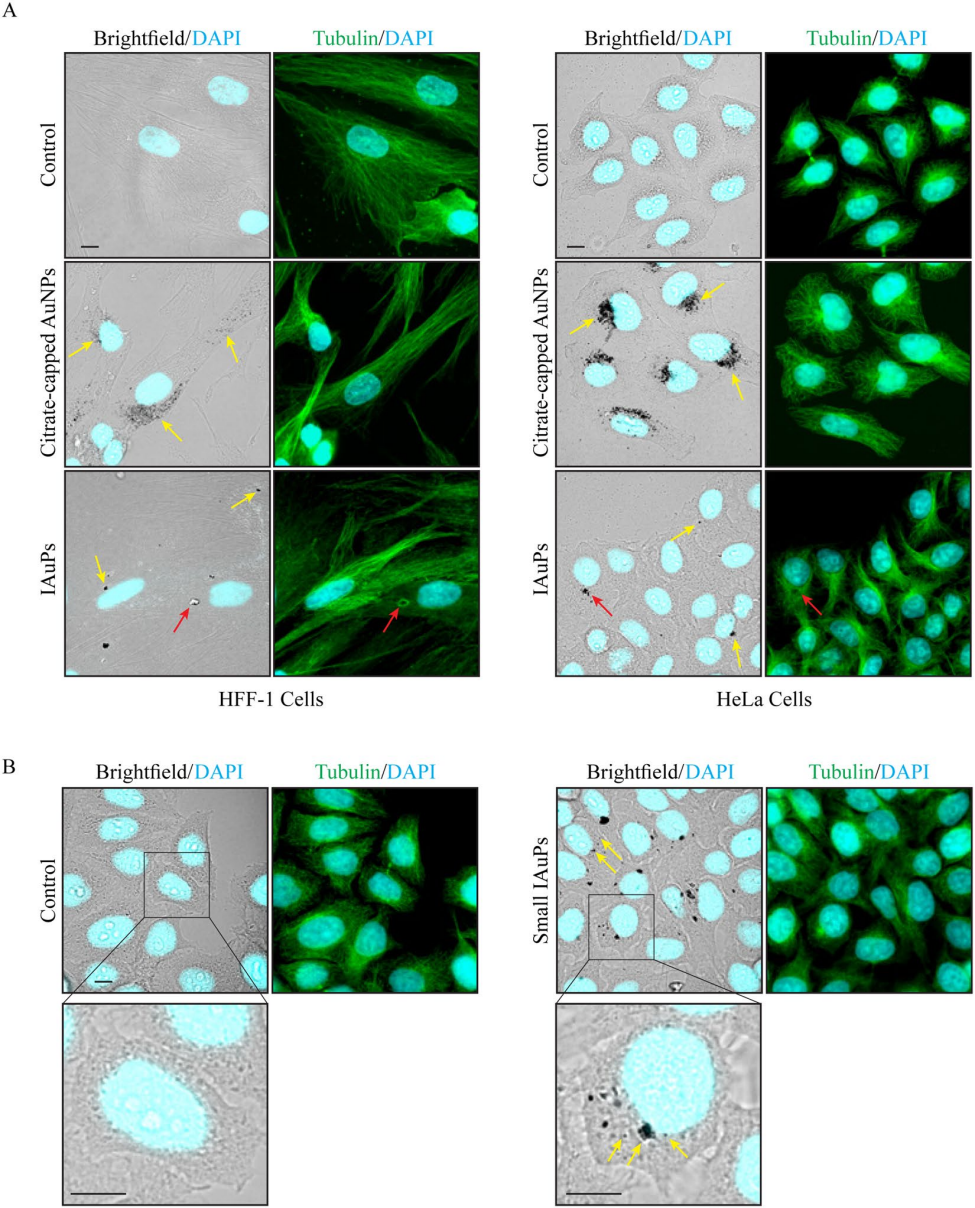
(**A**) Brightfield and fluorescence images of HFF-1 and HeLa cells co-stained for DAPI (to stain DNA; blue) and tubulin (to stain microtubules; green) show the location of AuNPs and IAuPs (yellow arrows). The red arrows point to microtubules that have been displaced around a particle. (**B**) Brightfield and fluorescence images of HeLa cells co-stained for DAPI (blue) and tubulin (green) show the location of mechanically disrupted IAuPs (small; yellow arrows). The scale bars are 10 μ*m*.

Since IAuPs have not been studied in human cells, their toxicity and subcellular location were characterized in comparison to AuNPs. Two well-characterized human cell lines were chosen for this study, namely HeLa cells, derived from human cervical adenocarcinoma, and HFF-1 (human foreskin fibroblasts) cells, which are non-cancerous. HeLa and HFF-1 cells were treated with citrate-capped AuNPs and IAuPs for 4 days. After, the cells were fixed and stained for tubulin, which is the core component of microtubules that controls cell architecture, and DAPI to visualize chromatin (Fig. 4). The brightfield and fluorescence microscopy images in Fig. 4A revealed that AuNPs accumulate in the endomembrane system surrounding the nucleus, which includes the golgi and endosomes. High levels of AuNPs accumulated in cells and appeared to be non-toxic, as no cell death was observed up to a week after treatment. Fewer cells had IAuPs, and their distribution was more varied in comparison to the AuNPs. While some of the particles appeared to be in the endomembrane system surrounding the nucleus, others were in vacuoles, cytosol, or in the nucleus as seen in Fig. 4A. Interestingly, some of the larger aggregates disrupted the microtubule networks, which appeared to ‘bend’ around them (Fig. 4B). Similar to the AuNPs, the IAuPs appeared to be non-toxic, as no cell death was observed up to a week after treatment.

To better compare IAuPs with AuNPs, the IAuPs were mechanically disrupted to break them into smaller particles (see schematic in Fig. 1). HeLa cells were treated with the smaller IAuPs as above, and imaged by light microscopy. The cells appeared to have more particles in comparison to the larger IAuPs (Fig. 4A vs. 4B). In addition, the smaller IAuPs accumulated in the endomembrane system similar to AuNPs (Fig. 4B). Thus, the larger size of the IAuPs is likely responsible for their different subcellular locations.

**Figure 5:**
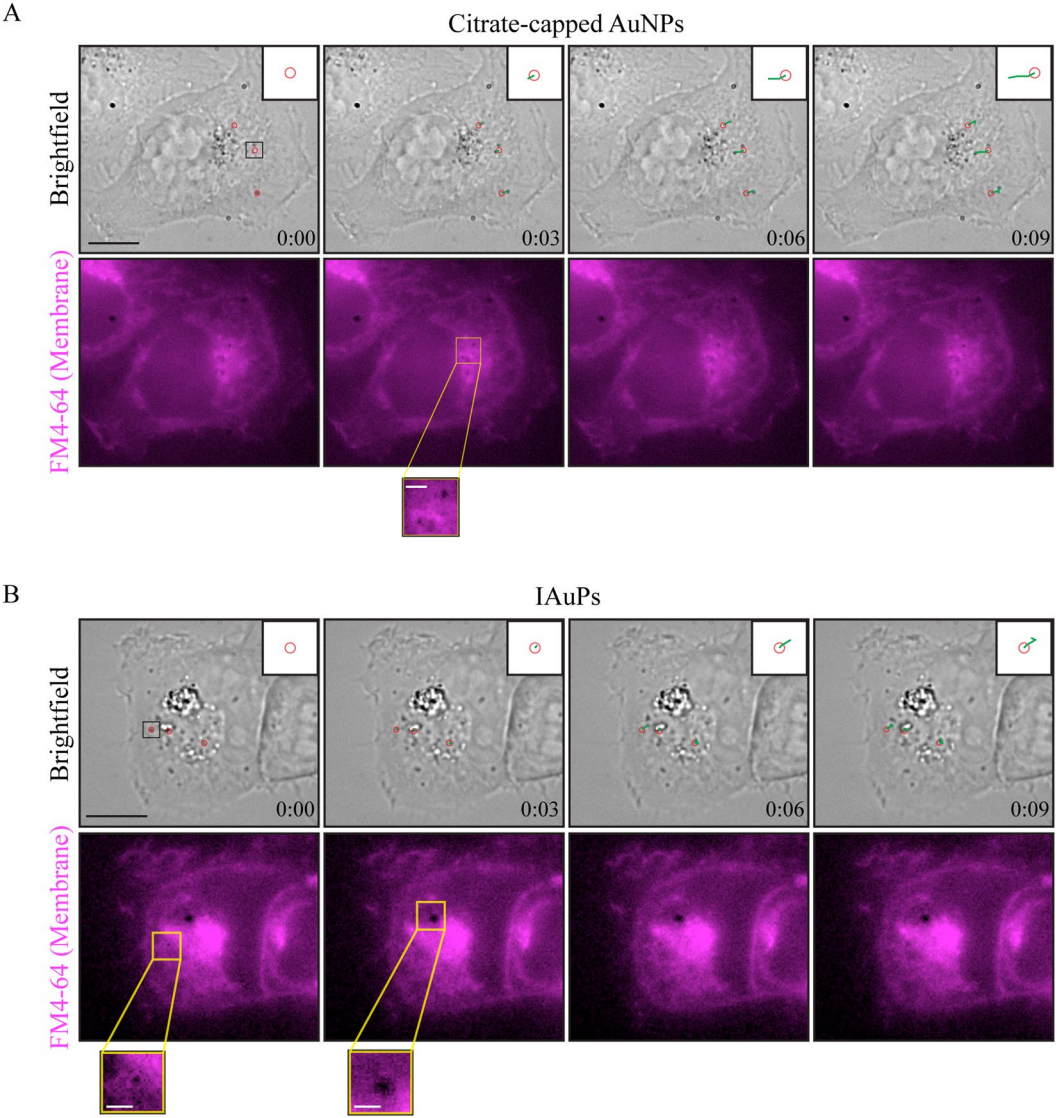
(**A**) Shown are time-lapse brightfield and fluorescence images of a HeLa cell treated with citrate-capped AuNPs and stained with FM 4-64 dye to show membranes. The inset in the top corner shows the start position of AuNPs (red circles) and the trajectory of their movement over time (green lines). The inset below shows a zoomed in image of particles surrounded by membrane. (**B**) Shown are time-lapse brightfield and fluorescence images of a HeLa cell treated with IAuPs and stained with FM 4-64 dye. The inset in the top corner shows the start position of IAuPs (red circles) and the trajectory of their movement over time (green lines). The lower insets show zoomed in images of a particle in a vesicle (left), and a particle that is not membrane-bound (right). The scale bars are 10 μ*m* for the cells and 2 μ*m* for the insets.

In order to obtain more information about the subcellular localization of AuNPs and IAuPs in HeLa cells, live imaging was performed using a membrane-specific dye (FM-4-64), after treatment with AuNPs or IAuPs (Fig. 5). Similar to fixed cells, AuNPs appeared to be localized to membrane-bound vesicles surrounding the nucleus as shown in Fig. 5. Tracking vesicle movement revealed trajectories that are consistent with movement along microtubule tracks, as expected for vesicles in the endomembrane system (green lines in Fig. 5). The localization of IAuPs was similar to what had been observed in fixed cells. Some particles appeared to be surrounded by membranes, while other particles were cytosolic, and their movement appeared to be random as shown in Fig. 5.

**Figure 6:**
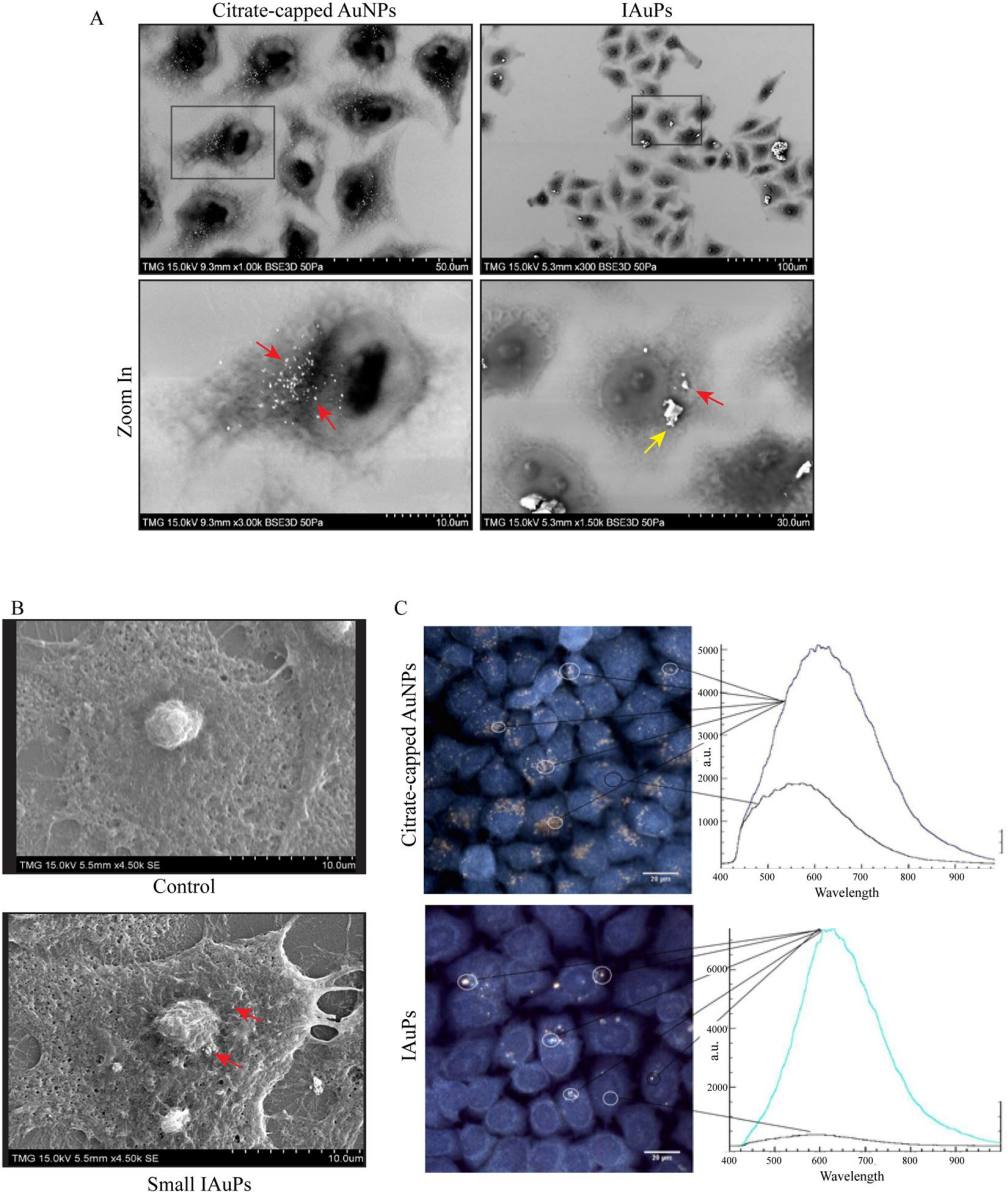
(**A**) The top panels show SEM images of IAuPs and citrate-capped AuNPs in HeLa cells. Single cells are shown at higher magnification, and the red arrow points to particles inside the cells, while the yellow arrow points to a particle outside of the cell. (**B**) SEM images show HeLa cells with either no-treatment (control) or after treatment with mechanically disrupted (small) IAuPs. The red arrows point to particles inside of the cell. (**C**) Cytoviva images of HeLa cells with IAuPs and citrate-capped AuNPs are shown. The corresponding spectral profiles for a selected particle vs. cytosol are shown for comparison. The scale is indicated for each image.

The physicochemical properties of AuNPs and IAuPs were characterized in cells. SEM of fixed HeLa cells after treatment with AuNPs revealed that, as expected, the particles were small and uniform in size, and had accumulated in the endomembrane system as indicated by the red arrows in Fig. 6A. However, IAuPs were larger and non-uniform in size as seen in Fig. 6A. Some of the larger particles had not entered the cells, as indicated by the shadow cast on cell marked by the yellow arrow, while others had entered the cells and were surrounded by membrane, or were in the cytosol (Fig. 6A). Imaging mechanically disrupted IAuPs in cells by SEM revealed that they were smaller and more uniform and size, and their location was similar to AuNPs, although there were fewer of them per cell (Fig. 6B).

Next, CytoViva imaging technology was used to compare the spectral profiles of AuNPs and IAuPs in HeLa cells using hyperspectral and dark-field illumination. As shown in Fig. 6C, the AuNPs had a broad peak between 600–700 nm, while IAuPs had a narrower peak between 600–625 nm. While the spectral shifts likely correspond to the difference in size between the two types of particles, differences in their spectral profiles also could be attributed to their interactions with different molecules inside the cells.

### Nuclear localization of IAuPs

**Figure 7:**
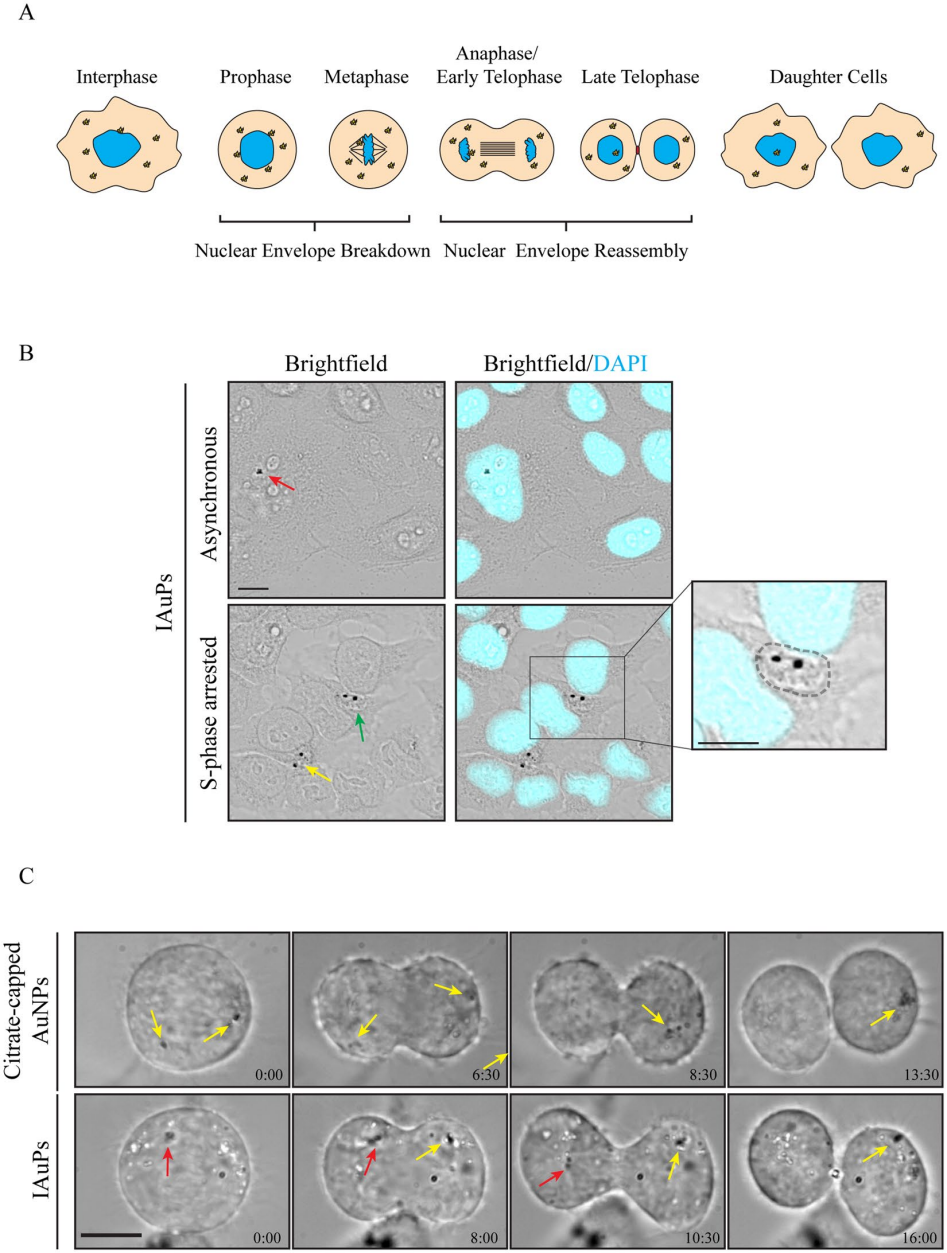
(**A**) A cartoon schematic shows the mechanism of nuclear entry for IAuPs during mitosis. As cells transition from prophase to metaphase, the nuclear envelope breaks down causing the nuclear and cytosolic contents to mix. The nuclear envelope reassembles in early telophase. (**B**) Images show asynchronous or S-phase arrested HeLa cells with IAuPs in the nucleus (red arrow), cytosol (yellow arrow) or in a vacuole (green arrow). The inset is a zoomed in region of a cell showing a vacuole, indicated by a dashed line, containing IAuPs. (**C**) Time-lapse images show dividing HeLa cells treated with citrate-capped AuNPs (top panel) or IAuPs (bottom panel). Yellow arrows indicate AuNPs or IAuPs that segregate to the poles of the cell, while red arrows point to IAuPs that stay near the chromatin and are likely incorporated into the nucleus. The scale bars are 10 μ*m*.

The nuclear localization of IAuPs was further investigated. Through functionalization, AuNPs can escape from lysosomes into the cytosol, where they could gain access to organelles such as mitochondria or the nucleus^30–34^. Although anti-cancer drugs like Doxorubicin can enter the nucleus without requiring a carrier, it may be desirable to use a carrier to have more control over drug targeting and/or release^39, 40^. AuNPs can be further modified so that after their escape into the cytosol, they can be selectively targeted to the nucleus^32^. However, particles in the cytosol could also randomly incorporate into the nucleus during division. As shown by the schematic in Fig. 7A, as cells enter mitosis to divide, their nuclear envelope breaks down, and nuclear components mix with the cytosol until they are re-packaged during telophase. IAuPs were observed in the nucleus of a small proportion of HeLa cells (Fig. 7B). To determine if IAuPs can be sequestered in the nucleus by chance during cell division, DIC microscopy was used to image dividing HeLa cells containing citrate-capped AuNPs or IAuPs (Fig. 7C). During mitosis, AuNPs remained closer to the cell poles as indicated by the yellow arrows in Fig. 7C. This is consistent with their retention in vesicles of the endomembrane system, which remain near the centrosomes during mitosis. The distribution and movement of IAuPs was random, as some particles remained close to the condensed chromatin, while others moved to opposite sides of the cell as shown in Fig. 7C. To verify that IAuPs enter the nucleus randomly during mitosis, HeLa cells were arrested in S phase to prevent them from entering mitosis. To ensure that the majority of cells in the population were synchronized for S phase, they were treated twice with 2 mM Thymidine^37^. After the second treatment, IAuPs were added to the cells, then after 23 hours were fixed and stained with DAPI to visualize chromatin. Brightfield and epifluorescence microscopy revealed that there were no IAuPs in the nuclei of cells that were arrested in S phase (0%; n = 200 cells, standard deviation = 0), while IAuPs were observed in the nuclei of a small subset of cells that were permitted to enter mitosis (1.6%, n = 193 cells, standard deviation = 0.25). When cells were left to cycle randomly for several generations, 10% had nuclear IAuPs. Therefore, this data suggests that IAuPs are randomly sequestered in the nucleus during nuclear envelope reformation after cell division rather than being selectively targeted.

### Entry mechanism of IAuPs

**Figure 8:**
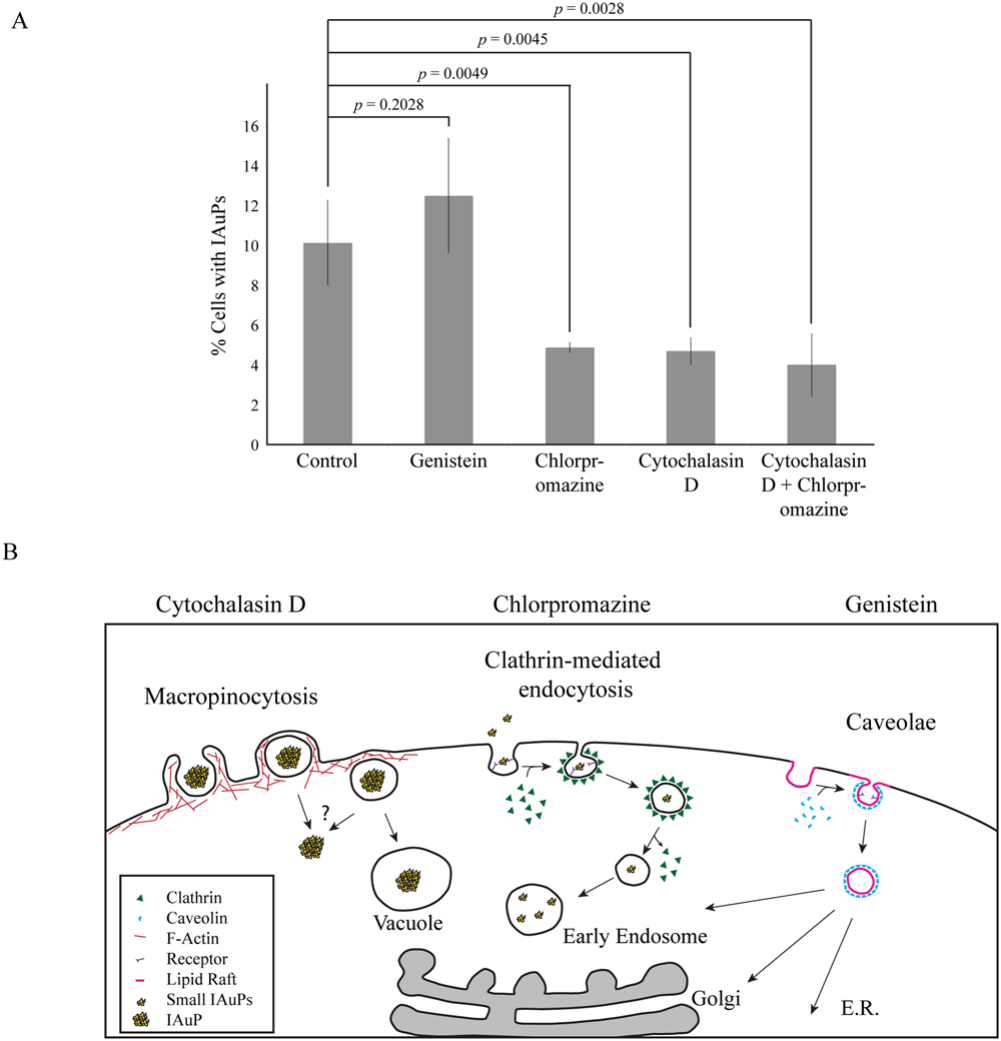
(**A**) The graph shows the proportion of cells with IAuPs after treatment with DMSO (control), 100 μ*M* Genistein, or 5 μ*M* Chlorpromazine or after treatment with 100 nM Cytochalasin D or both Cytochalasin D and Chlorpromorazine. Bars show standard deviation. (**B**) A cartoon schematic shows the different pathways by which the IAuPs enter HeLa cells, and those that are blocked by the drugs used in the assays in (**A**).

IAuPs differ from AuNPs in their physical properties and subcellular localization, and likely also vary in the mechanism by which they enter cells. Previous reports showed that AuNPs enter human cells via clathrin-dependent receptor-mediated endocytosis, where they remain in the endomembrane system^1, 21, 26, 28, 29^. While IAuP crystallites are 60 nm in size, they form large particles, and this study determined if IAuPs enter cells via macropinocytosis, clathrin-dependent receptor-mediated endocytosis, or clathrin-independent endocytosis^22–25^. As shown in Fig. 8A, HeLa cells treated with 100 nM Cytochalasin D to disrupt F-actin and block macropinocytosis had fewer IAuPs in comparison to control cells (4.7%, n = 727 cells vs. 9.2%, n = 628 cells for three replicates, respectively). Cells treated with 5 μ*m* Chlorpromazine, which disrupts clathrin-dependent receptor-mediated endocytosis also had fewer IAuPs compared to control cells (4.9%, n = 647 cells vs. 11.1%, n = 630 cells for three replicates, respectively; Fig. 8A). Cells treated with both Cytochalasin D and Chlorpromazine similarly showed a strong reduction in the number of cells with particles compared to control (4%, n = 643 cells vs. 9.2%, n = 628 cells for three replicates, respectively; Fig. 8A). Cells treated with 100 μ*M* Genistein to block caveolin-dependent endocytosis had no change in the number of particles compared to control cells (12.5%, n = 498 cells vs. 11.1%, n = 630 cells for three replicates, respectively; Fig. 8A). Thus, smaller IAuPs could enter cells via clathrin-dependent receptor-mediated endocytosis, while larger particles may rely more on macropinocytosis (Fig. 8B).

## Conclusions

This study describes the toxicity, subcellular distribution and entry mechanism of Swarna Bhasma Incinerated Gold Particles, IAuPs, in human cells. This is the first study, to our knowledge, that has characterized IAuPs in human cells. IAuPs are large irregular-shaped particles formed from 60 nm crystallites that are not toxic to HeLa or HFF-1 cells. As shown in Fig. 8B, small IAuPs likely enter HeLa cells via receptor-mediated endocytosis, where they accumulate in vesicles, within the endomembrane system, similar to AuNPs. However, large IAuPs may enter cells by macropinocytosis and accumulate in vacuoles. Vesicles that are part of the endomembrane system, display restricted patterns of movement, which mirrors that of the microtubule tracks they traffic along. Since microtubules, emanate from the centrosomes, the vesicles tend to accumulate in the endomembrane system that hugs the nucleus, where the centrosomes are located. In support of this, vesicles containing AuNPs showed these types of movements. However, while some vesicles with IAuPs showed these types of movements, others were more random. In addition, IAuPs were observed in the cytosol and nucleus in some cells, suggesting that they had escaped from vesicles or vacuoles.

The localization of IAuPs in the nucleus occurs during cell division. As eukaryotic cells enter mitosis, their nuclear envelope breaks down exposing chromatin to the cytosol, and the nuclear envelope re-assembles as chromosomes finish segregating during telophase. Particles that are in the cytosol are randomly located, and could get sequestered in one of the daughter nuclei as they reform. We propose that this may be a mechanism by which cytosolic particles gain access to the nucleus, which is often overlooked in the literature. Another interesting question is how IAuPs gain access to the cytosol when they all presumably enter via membrane-bound vesicles. As outlined above, IAuPs enter HeLa cells by receptor-mediated endocytosis and macropinocytosis, where they accumulate in membrane-bound vesicles or vacuoles, respectively. These membranous networks protect cells from foreign material and molecules. Large particles could cause membranes to break via mechanical disruption, or impede fission/fusion of the vesicles during remodeling of the membranous networks. Alternatively, IAuPs may contain elements or compounds that promote rupture of lysosomes or vacuoles. Once IAuPs gain access to the cytosol, they could be sequestered in the nucleus during division.

The composition of IAuPs varies between manufacturers even for those from a similar region. It is not clear how their composition impacts their entry, location or toxicity at the cellular level, or medicinal properties at the organismal level. It would be interesting to compare different IAuPs, as well as study their impact on different cell types than those studied here. For example, large particles may more successfully enter phagocytic cells (e.g. macrophages) causing an increase in the proportion of cells with IAuPs in comparison to HFF-1 or HeLa cells. IAuPs are typically administered by oral ingestion, and another question is how these particles are able to pass through the epithelial cells lining the digestive tract to enter the body, or if they enter the body at all and their medicinal properties are attributed to how they act as carriers for beneficial molecules from plant extracts. It would be interesting to explore the compounds and elements that are typically coupled with IAuPs to determine which ones confer medicinal properties. Some compounds could be small and amphipathic, permitting them to pass freely through the cell membranes. Given that IAuPs are inert, large particles, they could be further explored for use as carriers, imaging and/or temperature control for diagnostics or treatments.
